# The Institutional Library

**Published:** 1918-03-02

**Authors:** 


### THE INSTITUTIONAL LIBRARY.

**Syphilis and the Army.** By G. Thibierge. Edited by C. F. Marshall. (University of London Press. 6s. net.)

The University of London Press, Ltd., have issued, under the above title, another of the series of Military Medical Manuals it has undertaken. The book is a mine of information on the subject of this most fell disease, and will be of great value to all who seek information on the subject, whether doctors or sociologists. There are useful statistics in the book, which would be even better if the statistical portion had been more elaborated. The advice on social and personal preventative measures is sound common sense, but, reading them, one is convinced that nothing can be really effective to stop this curse of societies until compulsory notification is made the law. When syphilis is recognised to be a more dangerous disease to any nation than small-pox, and measures taken in accordance with this recognition, then there will be hope to stay the evil. As in the case of every disease that is not always amenable to treatment, there will be differences of opinion on the subject of treatment, so it is certain all will not agree with all that Dr. Thibièrge says on the subject, but the vast knowledge of the subject displayed in this book will compel respect, and as a guide to the less experienced the dissertations and hints given will be most useful. There is no question but that this book will be a most valuable addition to the library of every medical man who is ever called upon to treat syphilis—and how few are not? It is not only the vicious that suffer, as many passages in Dr. Thibièrge's book show.

**Serums, Vaccines, and Toxins.** By Wm. Cecil Bosanquet, M.A., M.D., and John W. H. Eyre, M.D. Third Edition. (London: Cassell and Co., Ltd. 1916. Pp. 445 + viii. Price 9s. net.)

At its first appearance this book broke new ground in the sense that it placed before the general body of the profession a systematic account of new methods both in diagnosis and in treatment. Success promptly awaited it, and necessitated a second edition. The present issue is a further tribute to its value. It deals with a department of medical knowledge which is continually enlarging its borders, and in some sections of which there is still much difference of opinion. The discussion of the whole subject is here presented with great fulness, and is brought well up to date. Completeness and thoroughness are two of its prominent features, and it may be consulted with every confidence. We congratulate the authors on the general recognition extended to their enterprise.

**Hygiene and Public Health.** By Sir Arthur Whitelegge, K.C.B., and Sir George Newman. Thirteenth Edition. (London: Cassell and Co., Ltd. 1917. Price 10s. 6d. net.)

By the time its thirteenth edition is required a book is so well established in favour and demand as to be beyond the reach of any ill-luck which superstition may attach to the number. Whitelegge and Newman's "Hygiene and Public Health," which is modestly described by the authors as an "introductory and elementary manual," has been twelve times revised since its publication twenty-seven years ago, a record which shows a determination to keep abreast of developments in the applications of preventive medicine. The science of hygiene has during that period undergone an extraordinary expansion both in knowledge and in its practical application. That this has not been arrested by the war but has been actually stimulated by it is shown by the present activity in the publication of books on hygiene, preventive medicine, the welfare of munition and other workers, and allied subjects. As Sir George Newman remarks in his preface to this edition, in the revision of which Sir Arthur Whitelegge has been unable to take any share, "in addition to a considerable growth in our knowledge of the etiology of disease and the conditions of its progress and prevention, there is an ever-widening sphere of influence in the State both for the voluntary and the official worker in preventive medicine."

This book is essentially a *vade mecum* for the medical officer of health. That it has been possible to keep it small enough to form one of Messrs. Cassell's excellent series of red-bound manuals is due primarily to careful condensation and abbreviation: the paper is thin and opaque and the printing excellent, and, while a judicious use is made of small type, diagrams and illustrations have been chosen sparingly. The book is not one which offers any attractions to the lay reader and as a text-book for the Public Health Diploma examinations is somewhat terse and condensed. We do not for a moment suggest that it is useless to the student, but that it is intended less for study than for reference; it is characterised by an absence of the disquisitive. Very important and excellent sections are those upon the duties of medical officers of health and sanitary inspectors; sanitary law, bye-laws and regulations; factories and workshops, the housing question, and house sanitation. Special chapters are devoted respectively to infection and immunity, the prevention of infectious diseases, and disinfection. The matters of maternity and infant welfare are not dealt with as their present importance and prominence demands, and the time has certainly come when the health visitor should be recognised and her duties defined; we find no reference to such an officer, even in connection with the Notification of Births (Extension) Act. This is an unfortunate omission in a work so practical, in which the general arrangement of subjects is so good, and which so clearly defines the powers and duties of the sanitary authority and the medical officer of health.

**Modern Methods in Nursing.** By Georgiana J. Sanders. Second Edition. (Philadelphia: W. B. Saunders and Co. $2.50 net.)

This book is, as its name implies, not so much a manual of nursing as a book of reference for the most modern forms of treatment. It is not a book suitable for junior probationers, but is rather intended for more advanced ones, who already understand the general nursing of different kinds of illness. Nurses studying for examinations, or those in charge of wards for the first time, would find the book useful when they wish to refer quickly to some special point. The index is arranged under symptoms and treatment. The directions about bedmaking are very full: and the author's advice, that nurses should be taught how to make hospital beds before they begin to work in the wards is very wise. It would be much easier for all parties if new probationers had some knowledge of this matter, though nothing but experience can teach them how to manage helpless cases. A dummy, or a fellow-probationer acting patient, is a very different thing from a really sick person. The

**THE INSTITUTIONAL LIBRARY**—(*continued*).

method of turning the mattress without removing the patient seems to be one that would cause him more exertion and strain on his nerves than lifting him gently on to another bed. Also, in the case of heavy patients, laying them on three pillows at one side of the bed, while the nurse pulls out the mattress and puts it back, would be very likely to end in a bad accident, if the nurses were not very strong and skilful, or if the patients were at all restless.

The method of giving all kinds of baths and packs is fully explained and illustrated. The author has evidently had a very unfortunate experience in the matter of hot bottles, as she directs that no operation patient is to have one in his bed without written orders from the doctor. Any properly trained nurse, or even any probationer after her first year, ought to be able to arrange hot bottles for her patient without burning him; provided, of course, that he is not left alone until the effects of the anæsthetic have passed off.

In the paragraphs treating of enemata it is rather a surprise to find the ball syringe even mentioned in a modern book. Its disadvantages are so many that it has been considered in most hospitals quite out of date. One finds, however, that there is often a kind of recurrent rhythm in treatment, and perhaps the ball syringe has had a temporary, if regrettable, resuscitation.

There is an exhaustive section on diet, and an appendix with a large number of recipes. Nurses should find this very useful when they are trying to give their patients variety in food. It might have been as well, however, to mention that there are still differences of opinion about the diet in typhoid fever. Even the patient's own opinion might cause some modification. It would often be impossible, especially in private cases, to induce patients in the acute stage of any fever to take fish, chicken, boiled eggs, buttered biscuits, and especially large quantities of sugar. Convalescent patients, of course, will usually take any quantity of food that is offered to them. The author's whole view of the nursing of typhoid hardly seems to allow of sufficient rest, either for the patient himself or for his digestive organs. An operation case was heard to remark lately that she had always thought the hours in illness must be so long and dreary, but that, on the contrary, she was thankful when she had a few minutes' peace, as there always seemed to be something to be done for her. This is not a very favourable state of mind in acute medical cases. It is an obvious retort that rest is not a "modern method": but this is not so true as some old-fashioned nurses would like to believe. The importance of rest is better understood now than it was some years ago. Many nurses will be glad of the help given by the large number of illustrations. It is sometimes very difficult to understand verbal descriptions of treatment, and a good picture will often make the matter much clearer.

**Health of the Munition Worker.** Handbook prepared by the Health of Munition Workers' Committee. (London: Published by H.M. Stationery Office. 1917. Pp. 138. Price 1s. 6d.)

An outline of the hygienic management of munition factories, giving the changes—generally in the direction of lightening the employee's task—which two years' experience has suggested. As in all public-health books, the treatment of the subject is, necessarily, extensive rather than intensive, a feature which precludes any review from taking the form of an "analysis." We note that no attempt has been made to introduce the speeding-up methods devised by psychologists; but perhaps the Ministry of Munitions has its hands full enough without these, and very likely no one on the Committee knew anything about them. Women police are not mentioned. Surely these have been employed where there are many female munitioneers?

**Modern Dietetics.** Feeding the Sick in Hospital and Home. With Some Studies on Feeding Well People. By Lulu Graves, Dietitian, Lakeside Hospital, Graveside. (St. Louis: The Modern Hospital Publishing Company. 1917.)

This well-thought-out volume is the outcome of some years' practical experience in catering for a large institution. The English reader wonders a little what sort of position the "dietitian" holds in the hospitals over the seas. Does she lecture on dietetics, or does she devote her entire time to the choice of the food-stuffs, their storage, their combination into diets, and their final preparation for the table? On all these matters Miss Graves appears to have a competent grip. The same difficulties over dishonest contractors and inadequate store-rooms beset her as beset the matron in many English institutions. But she has succeeded in bringing together two departments, the science and the art of feeding, which in England have till lately been hopelessly severed. For this reason her book should be very useful to domestic science students. Her remarks upon the food value of certain products, on milk and its modifications, on butter, cereals, eggs, vegetables and fruits, though containing nothing new, are clearly expressed, and deal with the best uses of these foods in institution dietetics as well as with their properties. The chapter on "Special Diets for Disease" is particularly helpful for nurses, and that on "Classes of People to be Fed" and "Feeding Various Institutions," for matrons. An excellent range of recipes is appended, and to most of these is added the number of "servings" produced by the given quantities, a very useful aid to the housekeeper. We are not sure that it is not rather waste of energy to work out the grams of protein, fat, and carbohydrates with the number of calories in "Hot Cross Buns," "Devil's Food Cake," and the like. But there is a craze for these reckonings at the moment, and they serve to demonstrate the nourishing properties of the dishes. Theories about the amount of proteins, etc., needed to build up the body are scattered to the winds under the pressure of war. They belong to the days when the cook could be light-heartedly directed to "take" flour and sugar, butter and cream in reckless abundance to make those delicate trifles which melt in the mouth. Such recipes read oddly in these days.

**The Medical Register.** (London: Published for the General Medical Council by Constable and Co., 10 Orange Street, Leicester Square. Pp. 1,198. Price 10s. 6d.)

The Register appears in the customary form, with the usual lists of British, Colonial, and foreign medical men in practice in this country, and the extracts from Acts of Parliament regulating the work of the medical profession.

**Our Girls in War Time.** Rhymes by Hampden Gordon, Pictures by Joyce Dennys, Authors of "Our Hospital A B C." (London: John Lane, The Bodley Head. Price 3s. 6d. net.)

These are spirited sketches of the war-time girl in some of her numerous attractive divagations and amazing costumes, pursued in most cases by the amorous glances of gentlemen in khaki. Nesta, the V.A.D., is a wicked little study taken from the life—perhaps.

